# Real-world outcomes of trifluridine/tipiracil with or without bevacizumab in refractory metastatic colorectal cancer: a multicenter cohort study from Turkey

**DOI:** 10.1007/s12672-026-05158-y

**Published:** 2026-05-08

**Authors:** Selami Bayram, Bahadir Koylu, Maral Martin Mildanoglu, Bilgin Demir, Hayati Arvas, Ozan Deniz Guven, Mustafa Serkan Alemdar, Tahir Yerlikaya, Fatih Selcukbiricik, Ahmet Bilici, Mukremin Uysal, Aysegul Kargi, Ali Murat Tatli, Muharrem Okan Cakir, Mustafa Ozdogan

**Affiliations:** 1 Department of Medical Oncology, Memorial Antalya Hospital, Antalya, Turkey; 2 Department of Internal Medicine, Division of Medical Oncology, Koç University School of Medicine, Istanbul, Turkey; 3 Department of Medical Oncology, Faculty of Medicine, Medipol University, Istanbul, 34083 Turkey; 4 Department of Internal Medicine, Division of Medical Oncology, Medical Faculty, Aydın Adnan Menderes University, Aydın, Turkey; 5 Department of Medical Oncology, Dicle University, Diyarbakir, Turkey; 6 Department of Medical Oncology, Antalya Training and Research Hospital, Antalya, Turkey; 7 Department of Medical Oncology, Medikal Park Antalya Hospital, Antalya, Turkey; 8 Department of Medical Oncology, Antalya City Hospital, Antalya, Turkey; 9 Department of Medical Oncology, Antalya Bilim University, Medstar Antalya Hospital, Antalya, Turkey; 10 School of Life Science, Pharmacy and Chemistry, Kingston University London, KT1 2EE 11 London, UK; 11 Department of Medical Oncology, Memorial Goztepe Hospital, Istanbul, Turkey

**Keywords:** Metastatic colorectal cancer, Trifluridine/tipiracil, FTD/TPI, TAS-102, Bevacizumab, Real-world, Retrospective, Overall survival, Progression-free survival

## Abstract

**Background:**

Trifluridine/tipiracil (FTD/TPI; TAS-102) is an established later-line treatment option for refractory metastatic colorectal cancer (mCRC), and randomized evidence supports the addition of bevacizumab. However, real-world data from Turkey are limited.

**Methods:**

We conducted a multicenter retrospective cohort study of adults with metastatic colorectal cancer (mCRC) who received FTD/TPI plus bevacizumab (combination) or FTD/TPI monotherapy in routine clinical practice between June 2021 and May 2025, with follow-up updated until September 25, 2025. Overall survival (OS) was the primary endpoint, and progression-free survival (PFS), response outcomes in radiologically evaluable patients, and safety were secondary endpoints. Survival was analyzed using Kaplan-Meier estimates and log-rank tests, with hazard ratios estimated using Cox regression. A prespecified multivariable Cox model for OS was adjusted for ECOG performance status, number of metastatic sites, liver metastasis, age group, and treatment group.

**Results:**

Seventy-eight patients were included (combination therapy, *n* = 57; monotherapy, *n* = 21). Median OS was 8 months (95% CI, 6.17–9.83) in the combination group and 6 months (95% CI, 5.03–6.97) in the monotherapy group (log-rank *p* = 0.437). Median PFS was 4 months (95% CI, 2.83–5.16) versus 3 months (95% CI, 1.92–4.07) (log-rank *p* = 0.409). Best radiologic response was evaluable in 65 patients. In univariable Cox regression, treatment group was not significantly associated with OS or PFS. In the multivariable Cox model for OS, treatment group remained not significantly associated with OS (adjusted HR 1.251, 95% CI 0.644–2.427; *p* = 0.509), whereas ECOG 1 (vs. 0) and liver metastasis (yes vs. no) were associated with worse OS. Hematologic toxicity was the dominant safety signal in both groups, and no unexpected safety signals were observed.

**Conclusions:**

In this multicenter Turkish real-world cohort, FTD/TPI-based therapy was feasible with manageable toxicity in heavily pretreated refractory mCRC. Comparative survival estimates between combination therapy and monotherapy were not statistically significant in unadjusted or adjusted analyses and should be interpreted as exploratory in light of baseline imbalances, subgroup-size asymmetry, and residual confounding. These findings complement, rather than challenge, the comparative efficacy estimates established in randomized trials such as SUNLIGHT.

**Supplementary Information:**

The online version contains supplementary material available at 10.1007/s12672-026-05158-y.

## Introduction

Metastatic colorectal cancer (mCRC) is a leading cause of cancer-related morbidity and mortality worldwide [1]. Approximately 15–30% of patients present with metastases at diagnosis, and 20–50% of initially localized cases eventually develop metastases [2]. Although outcomes have improved with the integration of combination fluoropyrimidine-based chemotherapy backbones (oxaliplatin- and irinotecan-containing regimens) and biologics targeting vascular endothelial growth factor (VEGF) or epidermal growth factor receptor (EGFR), most patients ultimately experience disease progression and require later-line systemic therapy [3]. Contemporary guidelines emphasize biomarker-driven options where actionable alterations exist (e.g., MSI-H/dMMR, BRAF V600E, HER2 amplification, and rare fusions). However, for most patients without targetable drivers, effective and tolerable salvage regimens remain central to routine clinical practice [4].

Trifluridine/tipiracil (FTD/TPI; TAS-102) is an oral cytotoxic agent that combines trifluridine, a thymidine-based nucleoside analog, with tipiracil, a thymidine phosphorylase inhibitor that increases trifluridine bioavailability [5]. FTD/TPI has become a standard option in refractory mCRC based on the phase 3 RECOURSE trial, which demonstrated a statistically significant overall survival (OS) benefit versus placebo with the best supportive care in heavily pretreated patients [6]. In parallel, regorafenib also established an OS benefit versus placebo in refractory disease, underscoring the clinical value of active late-line therapies, even when the response rates are modest [7].

Sustained angiogenesis inhibition beyond progression is a clinically relevant strategy in mCRC, as randomized studies have shown the benefit of continuing VEGF pathway blockade after the first progression [8, 9]. Preclinical colorectal cancer models further support the combination of FTD/TPI with bevacizumab, showing greater antitumor activity than either agent alone [10].

Before phase III confirmation, early phase and investigator-initiated studies evaluated FTD/TPI with bevacizumab. In the C-TASK FORCE phase 1/2 study, FTD/TPI plus bevacizumab demonstrated promising activity with manageable safety in a heavily pretreated population, meeting the primary activity endpoint (centrally assessed 16-week PFS) [11]. Subsequently, an investigator-initiated randomized phase 2 trial reported a clinically meaningful improvement in progression-free survival (PFS) with FTD/TPI plus bevacizumab versus FTD/TPI alone (median 4.6 vs. 2.6 months; HR 0.45) [12].

Real-world and retrospective cohorts have further suggested the effectiveness of this treatment in daily practice. In a Japanese retrospective cohort study (60 combination vs. 66 monotherapy), the addition of bevacizumab improved PFS (median 3.7 vs. 2.2 months (HR 0.69) with manageable toxicity [13]. A retrospective study using propensity score matching reported a longer overall survival with FTD/TPI plus bevacizumab than with FTD/TPI alone, although observational comparisons remain susceptible to selection bias and residual confounding [14].

However, real-world data from non-Asian populations are limited. A Spanish real-world cohort of FTD/TPI plus bevacizumab reported outcomes broadly consistent with prior clinical and observational evidence (median PFS, 4.3 months; median OS, 9.3 months) and supported its feasibility in routine practice [15].

The phase 3 SUNLIGHT trial subsequently provided high-level evidence to support this strategy. In the SUNLIGHT trial, patients with refractory mCRC randomized to receive FTD/TPI plus bevacizumab versus FTD/TPI alone experienced significant improvements in OS (median 10.8 vs. 7.5 months; HR 0.61), and PFS (median 5.6 vs. 2.4 months (HR 0.44), with no treatment-related deaths and a safety profile consistent with the known toxicities of the component agents, including bevacizumab-related adverse events such as hypertension [16].

Despite the growing international evidence base, region-specific real-world data remain valuable because treatment sequencing, supportive care, and access to later-line therapies vary across different health systems. In this study, we evaluated the real-world effectiveness and safety of FTD/TPI with or without bevacizumab in patients with refractory metastatic colorectal cancer treated in routine clinical practice.

## Materials and methods

### Study design and setting

This multicenter, retrospective cohort study evaluated the effectiveness and safety of trifluridine/tipiracil (FTD/TPI; TAS-102) administered with bevacizumab versus FTD/TPI monotherapy in patients with metastatic colorectal cancer (mCRC) treated in routine clinical practice in Turkey.

### Study population

Adult patients (≥ 18 years) with histologically confirmed colorectal adenocarcinoma and unresectable locally advanced or metastatic disease were eligible if they received FTD/TPI-based therapy (FTD/TPI plus bevacizumab or FTD/TPI monotherapy) for mCRC at participating centers. Eligible patients were those who initiated FTD/TPI-based therapy between June 2021 and May 2025. Follow-up was updated until September 25, 2025, which served as the data cut-off. The index date for time-to-event analyses was defined as the date of treatment initiation (first dose) of FTD/TPI-based therapy. Adequate clinical documentation was required to ascertain treatment exposure and outcomes, including at least one follow-up record after treatment initiation. Patients treated with FTD/TPI for non-colorectal histologies or lacking essential data required for time-to-event evaluation (treatment start date and vital status follow-up) were excluded from the study. Given the retrospective design, no prospective sample size calculation was performed; instead, all eligible patients treated during the study period were included in the analysis.

### Data sources and variables

Data were abstracted from electronic medical records and institutional oncology databases using a pre-defined data collection framework. Extracted variables included demographic characteristics (age and sex), baseline clinical features (ECOG performance status, primary tumor location, and sidedness where available, de novo metastatic status), disease burden (number of metastatic sites and organ-specific involvement), molecular features (RAS, BRAF, MSI/MMR, and HER2 when available), prior systemic therapy exposures (fluoropyrimidine, oxaliplatin, irinotecan, anti-VEGF therapy, anti-EGFR therapy, regorafenib), and baseline laboratory parameters when available. Safety data were obtained from clinical notes and laboratory records. Adverse events were summarized by type and grade; when CTCAE grading was not explicitly documented, severity was recorded as described in the medical record and categorized to the closest corresponding grade, when feasible.

### Treatment exposure

Patients were categorized according to treatment regimen: FTD/TPI plus bevacizumab (combination) or FTD/TPI monotherapy. FTD/TPI and bevacizumab were administered in accordance with approved labels and local institutional practices. Dose modifications, delays, and discontinuations were performed at the discretion of the treating physician based on toxicity and clinical status. Treatment allocation was not protocol-mandated. In routine practice, the choice between FTD/TPI plus bevacizumab and FTD/TPI monotherapy was made by the treating physicians based on overall clinical judgment, including performance status, metastatic burden, prior treatment exposure, comorbidity profile, and the perceived suitability for bevacizumab-containing therapy. The reason for treatment discontinuation (e.g., progression, toxicity) was recorded when applicable.

### Outcomes

The primary endpoint was overall survival (OS), defined as the time from initiation of FTD/TPI-based therapy (index date) to death from any cause. Patients alive at the last contact were censored on the date of the last known follow-up. Secondary endpoints included progression-free survival (PFS), defined as the time from the index date to the first documented disease progression or death from any cause, whichever occurred first.

PFS reflected investigator-assessed progression in routine clinical practice. Progression was primarily based on radiologic evaluation when follow-up imaging was available; clinical progression was also accepted when explicitly documented by the treating physician in the absence of formal radiologic progression documentation. Imaging intervals were not centrally standardized across participating centers and generally reflected local routine follow-up practice. Additional secondary endpoints included investigator-assessed tumor response and disease control among radiologically evaluable patients.

Tumor response was retrospectively abstracted from imaging reports and treating-physician documentation. RECIST version 1.1 principles were applied where measurable disease and sufficiently detailed imaging documentation were available; however, no centralized radiologic review was performed. Radiologically evaluable patients were defined as those with available post-baseline imaging and sufficient documentation to permit retrospective response categorization in routine clinical practice. Patients without adequate follow-up imaging or without sufficient radiologic documentation for retrospective response classification were not included in the response analysis. Safety and tolerability were assessed by adverse events and laboratory abnormalities recorded during treatment.

### Statistical analysis

Descriptive statistics are presented as median (interquartile range [IQR]) for continuous variables and number (percentage) for categorical variables. Between-group comparisons were performed using the Mann-Whitney U test for continuous variables and the chi-square test or Fisher’s exact test for categorical variables, as appropriate. Overall survival (OS) and progression-free survival (PFS) were estimated using the Kaplan-Meier method and compared using the log-rank test. Hazard ratios (HRs) with 95% confidence intervals (CIs) were estimated using Cox proportional hazards models. In addition to univariable models, a prespecified multivariable Cox model for OS was fitted. Covariates were selected a priori based on clinical relevance, established prognostic importance in metastatic colorectal cancer, and data completeness, rather than by automated statistical selection procedures. To reduce the risk of overfitting in a modest-sized cohort, a parsimonious model was used. The final model included ECOG performance status (0 vs. 1), number of metastatic sites (1, 2, ≥ 3), liver metastasis (no vs. yes), age (< 65 vs. ≥ 65), and treatment group (combination vs. monotherapy; reference = monotherapy).

Propensity-based approaches, including propensity score matching, inverse probability weighting, and propensity-adjusted Cox models, were considered. However, these methods were not pursued because of the modest sample size, the relatively small monotherapy subgroup, and concerns regarding limited covariate overlap, unstable estimates, and model overfitting in a substantially imbalanced retrospective cohort. The proportional hazards assumption was assessed using log(-log) survival plots and time-dependent checks, where appropriate. All tests were two-sided, and *p* < 0.05 was considered statistically significant. Analyses were conducted using SPSS version 23.0.

## Results

### Study population

A total of 78 patients with metastatic colorectal cancer were included in this study. FTD/TPI plus bevacizumab was administered to 57 patients (73.1%), and FTD/TPI monotherapy was administered to 21 patients (26.9%) (Tables 1, 2). Best radiologic response was evaluable in 65 patients (46 and 19 in the combination and monotherapy groups, respectively) (Table 3). The remaining 13 patients were not included in the response analysis because adequate post-baseline imaging or sufficiently detailed radiologic documentation for retrospective response categorization was unavailable, typically in the setting of early progression, clinical deterioration, or limited follow-up before formal reassessment.

**Table 1:** Baseline characteristics of patients with refractory metastatic colorectal cancer treated with FTD/TPI plus bevacizumab or FTD/TPI monotherapy

Characteristic	Overall (*n* = 78)	FTD/TPI plus Bevacizumab (*n* = 57)	FTD/TPI (*n* = 21)	*P* value
**Age, years**				
— Median (Q1–Q3), years	63 (54–71)	62.5 (51.5–72)	63 (55–67)	0.736
— <65 years	46 (59.0)	32 (56.1)	14 (66.7)	0.402
— ≥65 years	32 (41.0)	25 (43.9)	7 (33.3)	0.402
**Sex**,** n (%)**				0.437
— Female	28 (35.9)	19 (33.3)	9 (42.9)	
— Male	50 (64.1)	38 (66.7)	12 (57.1)	
**ECOG performance-status score**,** n (%)**				0.006
— 0	49 (62.8)	41 (71.9)	8 (38.1)	
— 1	29 (37.2)	16 (28.1)	13 (61.9)	
**Primary diagnosis**,** n (%)**				0.842
— Colon cancer	46 (59.0)	34 (59.6)	12 (57.1)	
— Rectum cancer	32 (41.0)	23 (40.4)	9 (42.9)	
**Location of primary tumor**,** n (%)**				0.213
— Right-sided	15 (19.2)	9 (15.8)	6 (28.6)	
— Left-sided (incl. rectum)	63 (80.8)	48 (84.2)	15 (71.4)	
**De novo metastatic disease**,** n (%)**				0.353
— No	27 (34.6)	18 (31.6)	9 (42.9)	
— Yes	51 (65.4)	39 (68.4)	12 (57.1)	
**Number of metastatic sites**,** n (%)**				0.004
— 1	15 (19.2)	14 (24.6)	1 (4.8)	
— 2	20 (25.6)	18 (31.6)	2 (9.5)	
— ≥3	43 (55.1)	25 (43.9)	18 (85.7)	
Metastatic sites*, **n (%)**				
— Liver	62 (79.5)	46 (80.7)	16 (76.2)	0.754
— Lung	55 (70.5)	37 (64.9)	18 (85.7)	0.074
— Peritoneum	25 (32.1)	16 (28.1)	9 (42.9)	0.215
— Bone	23 (29.5)	16 (28.1)	7 (33.3)	0.651
— Non-regional lymph node	41 (52.6)	24 (42.1)	17 (81.0)	0.002
**Primary tumor resected**,** n (%)**				0.173
— No	24 (30.8)	20 (35.1)	4 (19.0)	
— Yes	54 (69.2)	37 (64.9)	17 (81.0)	
**RAS status**,** n (%)**				0.564
— Wild-type	33 (42.3)	23 (40.4)	10 (47.6)	
— Mutant	45 (57.7)	34 (59.6)	11 (52.4)	
**BRAF status**,** n (%)**				0.558
— Wild-type	75 (96.2)	54 (94.7)	21 (100.0)	
— Mutant	3 (3.8)	3 (5.3)	0 (0.0)	
**MSI/MMR status**,** n (%)**				1
— pMMR/MSS or MSI-L	71 (91.0)	51 (89.5)	20 (95.2)	
— dMMR/MSI-H	1 (1.3)	1 (1.8)	0 (0.0)	
— Not tested/unknown	6 (7.7)	5 (8.8)	1 (4.8)	
**HER2 status**,** n (%)**				0.165
— Negative	60 (76.9)	43 (75.4)	17 (81.0)	
— Positive	5 (6.4)	2 (3.5)	3 (14.3)	
— Not tested/unknown	13 (16.7)	12 (21.1)	1 (4.8)	
**Palliative treatment line**,** n (%)**				0.183
— Second line	6 (7.7)	6 (10.5)	0 (0.0)	
— Third line or later	72 (92.3)	51 (89.5)	21 (100.0)	
**Previous treatments received for metastatic disease**,** n (%)**				
— Fluoropyrimidine	77 (98.7)	56 (98.2)	21 (100.0)	1
— Irinotecan	77 (98.7)	56 (98.2)	21 (100.0)	1
— Oxaliplatin	75 (96.2)	54 (94.7)	21 (100.0)	0.559
— Anti-VEGF monoclonal antibody	70 (89.7)	54 (94.7)	16 (76.2)	0.029
— Anti-EGFR monoclonal antibody	34 (43.6)	23 (40.4)	11 (52.4)	0.342
— Regorafenib	66 (84.6)	45 (78.9)	21 (100.0)	0.03

p values were calculated using the Mann–Whitney U test for continuous variables and chi-square/Fisher’s exact test for categorical variables, as appropriate; two-sided p < 0.05 was considered significant. *, Metastatic-site categories are not mutually exclusive. For MSI/MMR and HER2, ‘Not tested/unknown’ is reported as a separate category, and percentages are calculated using the total number of patients in each column. MSI/MMR status was available for 72/78 patients; HER2 status was available for 65/78 patients

**Table 2:** Clinical outcomes in patients treated with FTD/TPI plus bevacizumab versus FTD/TPI monotherapy

Outcome	FTD/TPI plus Bevacizumab (*n* = 57)	FTD/TPI (*n* = 21)	*P* value
**Overall survival (OS)**			
Median OS, months (95% CI)	8 (6.17–9.83)	6 (5.03–6.97)	0.437 (log-rank)
6-month OS, %	62.7	41.2	—
12-month OS, %	34.2	29.4	—
Univariable Cox HR (95% CI)	0.804 (0.45–1.43)	Reference	0.460
**Progression-free survival (PFS)**			
Median PFS, months (95% CI)	4 (2.83–5.16)	3 (1.92–4.07)	0.409 (log-rank)
6-month PFS, %	22.8	23.7	-
12-month PFS, %	2.7	17.8	-
Univariable Cox HR (95% CI)	1.23 (0.70–2.14)	Reference	0.465

Median overall survival (OS) and progression-free survival (PFS) were estimated using the Kaplan–Meier method and are presented with 95% confidence intervals (CIs). The P values for median OS and PFS correspond to two-sided log-rank tests. Six- and 12-month OS/PFS rates represent Kaplan–Meier estimates. Hazard ratios (HRs) with 95% CIs were derived from univariable Cox proportional hazards models comparing FTD/TPI plus bevacizumab with FTD/TPI monotherapy (reference**)**. OS, overall survival; PFS, progression-free survival; CI, confidence interval; HR, hazard ratio. -, not applicable,

**Table 3:** Best radiologic response to FTD/TPI-based therapy by treatment group among evaluable patients (*n* = 65)

Best response	FTD/TPI plus Bevacizumab (*n* = 46)	FTD/TPI monotherapy (*n* = 19)	Overall (*n* = 65)
Complete response (CR)	0 (0.0)	1 (5.3)	1 (1.5)
Partial response (PR)	8 (17.4)	1 (5.3)	9 (13.8)
Stable disease (SD)	4 (8.7)	6 (31.6)	10 (15.4)
Progressive disease (PD)	34 (73.9)	11 (57.9)	45 (69.2)
**Objective response rate (ORR)**	**8/46 (17.4)**	**2/19 (10.6)**	**10/65 (15.4)**
**Disease control rate (DCR)**	**12/46 (26.1)**	**8/19 (42.2)**	**20/65 (30.8)**

Response was assessed per RECIST (version 1.1) principles based on available imaging in routine clinical practice. Percentages were calculated within each treatment group among the evaluable patients. ORR was defined as a complete response (CR) plus partial response (PR). DCR was defined as CR plus PR plus stable disease (SD). FTD/TPI, trifluridine/tipiracil

**Table 4:** Multivariable Cox proportional hazards regression for overall survival (OS)

Covariate	Comparison (reference)	Adjusted HR (95% CI)	*P* value
ECOG performance status	ECOG 1 vs. ECOG 0	2.214 (1.208–4.059)	0.010
Number of metastatic sites (overall)	—	—	0.338
	2 vs. 1	0.673 (0.295–1.537)	0.347
	≥ 3 vs. 1	1.203 (0.592–2.444)	0.609
Liver metastasis	Yes vs. No	2.593 (1.113–6.043)	0.027
Treatment	Combination vs. Monotherapy	1.251 (0.644–2.427)	0.509
Age	≥ 65 vs. < 65	0.716 (0.406–1.264)	0.250

HR, hazard ratio; CI, confidence interval; ECOG, Eastern Cooperative Oncology Group. Reference categories: ECOG 0; number of metastatic sites = 1; no liver metastasis; monotherapy group; age < 65 years. The P value for “Number of metastatic sites (overall)” corresponds to the omnibus (global) test for that variable. Two-sided P values

### Baseline demographic and clinical characteristics

The baseline characteristics are summarized in Table 1. The median age was 63 years (IQR, 54–71), and 46 patients (59.0%) were < 65 years old. The majority were male (64.1%), and all patients had an ECOG performance status of 0–1 (ECOG 0: 62.8%; ECOG 1: 37.2%) (Table 4).

Primary tumors originated from the colon (59.0%) or rectum (41.0%), with predominantly left-sided or rectal disease (80.8%). De novo metastatic disease was present in 65.4% of patients, and ≥ 3 metastatic sites were documented in 55.1% of patients. The most frequent metastatic sites were the liver (79.5%) and lung (70.5%), followed by non-regional lymph nodes (52.6%), peritoneum (32.1%), and bones (29.5%) (Table 1). Primary tumor resection was performed in 69.2% of the patients.

Molecular profiling showed RAS mutations in 57.7% of patients and BRAF mutations in 3.8% of patients. MSI/MMR status was available for 72/78 patients (92.3%); one patient (1.4% of those tested) had dMMR/MSI-H, while 6/78 patients (7.7%) were not tested or had unknown results. HER2 status was available for 65/78 patients (83.3%); HER2 positivity was observed in five patients (7.7% of those tested), while 13/78 patients (16.7%) were not tested or unknown (Table 1).

Most patients initiated FTD/TPI -based treatment in later lines: 92.3% received therapy in the third line or beyond, and only six patients (7.7%) were treated in the second-line setting (Table 1). Prior exposure to standard mCRC agents was common, including fluoropyrimidine (98.7%), irinotecan (98.7%), oxaliplatin (96.2%), anti-VEGF therapy (89.7%), anti-EGFR therapy (43.6%), and regorafenib (84.6%) (Table 1). These findings confirm that the cohort represented a heavily pretreated real-world population. Between-group differences in prior treatment history were also observed, further supporting the interpretation that treatment allocation reflected real-world clinical selection rather than baseline comparability.

### Between-group baseline differences

Several clinically relevant imbalances were observed between the treatment groups (Table 1). Compared with the combination therapy group, the monotherapy group had a higher proportion of ECOG 1 patients (61.9% vs. 28.1%; *p* = 0.006), a substantially higher proportion of patients with ≥ 3 metastatic sites (85.7% vs. 43.9%; *p* = 0.004), and more frequent non-regional lymph node metastases (81.0% vs. 42.1%; *p* = 0.002). Prior anti-VEGF exposure was lower (76.2% vs. 94.7%; *p* = 0.029), whereas prior regorafenib exposure was higher (100.0% vs. 78.9%; *p* = 0.030) in the monotherapy group. These imbalances were considered when interpreting the comparative outcomes.

### Treatment exposure and discontinuation

Detailed treatment-exposure metrics, such as the exact number of FTD/TPI cycles and the total number of bevacizumab administrations, were not available in a sufficiently standardized format across centers for robust pooled reporting. However, treatment discontinuation reason was available for a substantial proportion of patients.

Among patients with a documented reason for discontinuation, disease progression was the predominant cause in both treatment groups. In the combination group, 43 of 44 patients (97.7%) with available discontinuation data stopped treatment because of progression, whereas 1 patient (2.3%) discontinued because of toxicity. In the monotherapy group, 16 of 17 patients (94.1%) with available discontinuation data discontinued because of progression, and 1 patient (5.9%) discontinued due to toxicity. These findings indicate that treatment cessation in routine practice was driven primarily by disease progression rather than adverse events (Supplementary Table S2).

### Tumor response

Best radiologic response was evaluable in 65 of 78 patients (46/57 in the combination group and 19/21 in the monotherapy group). The remaining 13 patients were not included in the response analysis because adequate post-baseline imaging or sufficiently detailed radiologic documentation for retrospective response categorization was unavailable, typically in the setting of early progression, clinical deterioration, or limited follow-up before formal reassessment.

The ORR was 17.4% and 10.6% in the FTD/TPI plus bevacizumab and FTD/TPI monotherapy groups, respectively, whereas the DCRs were 26.1% and 42.2%, respectively (Table 3). This pattern appears to reflect different response-category distributions rather than contradictory treatment signals: the combination group had a higher proportion of partial responses, while the monotherapy group had a higher proportion of stable disease among evaluable patients. In light of the small evaluable subset, non-random treatment allocation, and baseline imbalances, these response findings should be interpreted descriptively and as hypothesis-generating.

### Overall survival

Overall survival (OS) was the primary outcome. Median OS was 8 months (95% CI, 6.17–9.83) in the FTD/TPI plus bevacizumab group and 6 months (95% CI, 5.03–6.97) in the FTD/TPI monotherapy group. The Kaplan-Meier curves did not differ significantly (log-rank *p* = 0.437).

In univariable Cox regression, treatment group was not significantly associated with mortality (HR 0.804, 95% CI 0.45–1.43; *p* = 0.460; combination vs. monotherapy).

In multivariable Cox regression adjusting for ECOG performance status, number of metastatic sites, liver metastasis, and age, treatment group remained not significantly associated with OS (adjusted HR 1.25, 95% CI 0.644–2.427; *p* = 0.509) with monotherapy as the reference group. In this model, ECOG 1 (vs. 0) was associated with worse OS (adjusted HR 2.214, 95% CI 1.208–4.059; *p* = 0.010), and liver metastasis (yes vs. no) was also associated with worse OS (adjusted HR 2.593, 95% CI 1.113–6.043; *p* = 0.027). The apparent change in direction between the univariable and multivariable treatment hazard ratios should be interpreted with substantial caution, as it likely reflects baseline imbalance, correlation among prognostic covariates, limited effective sample size, and instability of regression estimates in a modest-sized observational cohort. Therefore, the adjusted treatment coefficient should be viewed as an exploratory model-based estimate rather than a definitive measure of comparative effectiveness.

### Progression-free survival

Progression-free survival (PFS) was analyzed as the secondary endpoint. Median PFS was 4 months (95% CI, 2.83–5.16) in the combination group and 3 months (95% CI, 1.92–4.07) in the monotherapy group. No statistically significant difference was observed (log-rank *p* = 0.409).

In univariable Cox regression, treatment group was not significantly associated with the risk of progression or death (HR 1.23, 95% CI 0.70–2.14; *p* = 0.465) with monotherapy as the reference group.

### Safety

Adverse events were predominantly hematologic and were generally consistent with the known safety profile of FTD/TPI-based therapy. When analyzed by treatment group, hematologic toxicity remained the dominant adverse-event category in both cohorts (Table 5).

**Table 5:** Treatment-group-stratified adverse events in patients receiving FTD/TPI plus bevacizumab or FTD/TPI monotherapy

Adverse event	Combination therapy, *n* (%) Any grade/Grade ≥ 3	Monotherapy, *n* (%) Any grade/Grade ≥ 3
Anemia	16 (28.1)/4 (7.0)	3 (14.3)/2 (9.5)
Leukopenia	11 (19.3)/4 (7.0)	6 (28.6)/2 (9.5)
Neutropenia	10 (17.5)/5 (8.8)	7 (33.3)/4 (19.0)
Thrombocytopenia	10 (17.5)/2 (3.5)	2 (9.5)/1 (4.8)
Nausea	12 (21.1)/0	1 (4.8)/0
Fatigue	25 (43.9)/1 (1.8)	8 (38.1)/0

Percentages are calculated using the total number of patients in each treatment group as the denominator

In the combination group (*n* = 57), any-grade anemia, leukopenia, neutropenia, and thrombocytopenia occurred in 28.1%, 19.3%, 17.5%, and 17.5% of patients, respectively; corresponding grade ≥ 3 rates were 7.0%, 7.0%, 8.8%, and 3.5%. In the monotherapy group (*n* = 21), any-grade anemia, leukopenia, neutropenia, and thrombocytopenia occurred in 14.3%, 28.6%, 33.3%, and 9.5% of patients, respectively; corresponding grade ≥ 3 rates were 9.5%, 9.5%, 19.0%, and 4.8% (Table 5).

Non-hematologic toxicities remained mostly low in both groups. No unexpected safety signals were observed. Given the retrospective design and modest sample size, these safety findings should be interpreted descriptively.

## Discussion

In this multicenter Turkish real-world cohort of heavily pretreated metastatic colorectal cancer (mCRC), we describe the outcomes and safety of trifluridine/tipiracil (FTD/TPI) administered with or without bevacizumab in routine clinical practice. Three messages are clinically relevant in this study. First, FTD/TPI-based therapy was deliverable in this setting, with a toxicity profile consistent with prior evidence. Second, survival outcomes were within the range expected for later-line mCRC. Third, while unadjusted median overall survival numerically favored the combination (8 vs. 6 months) and median progression-free survival was modestly longer (4 vs. 3 months), between-group differences were not statistically significant, and the observational design limited causal interpretation.

Our findings should be interpreted alongside the established randomized evidence for FTD/TPI and bevacizumab intensification in refractory disease. The phase III RECOURSE trial established FTD/TPI as an effective later-line option, demonstrating an overall survival benefit versus placebo in refractory mCRC with predictable myelosuppression as the dominant toxicity [6]. The strategy of adding bevacizumab to FTD/TPI was subsequently supported by prospective evidence, including the C-TASK FORCE phase I/II study and a randomized phase II trial by Pfeiffer et al., which reported a clinically meaningful improvement in progression-free survival with combination therapy [11, 12]. Most importantly, the phase III SUNLIGHT trial demonstrated significant improvements in both overall survival and progression-free survival for FTD/TPI plus bevacizumab versus FTD/TPI alone, establishing the combination as an evidence-based later-line treatment option [16]. Accordingly, the present study is not intended to re-estimate or challenge the treatment effect established in SUNLIGHT. Rather, its principal contribution is to provide region-specific real-world data regarding the feasibility, tolerability, and implementation of FTD/TPI-based therapy in routine Turkish clinical practice, where patient selection, treatment sequencing, access conditions, and supportive care patterns may differ from those in prospective clinical trials. In this context, the absence of statistically significant between-group differences in our cohort should be interpreted primarily as a reflection of real-world heterogeneity, limited sample size, and residual confounding, rather than as evidence against the benefit of combination therapy shown in randomized settings.

Several aspects of routine practice likely influenced comparative estimates in this retrospective dataset. Treatment allocation was non-random, and clinicians may preferentially offer bevacizumab-containing therapy to patients perceived as more likely to tolerate combination treatment while reserving monotherapy for patients with less favorable baseline characteristics, greater metastatic burden, or lower suitability for bevacizumab-containing therapy. This confounding by indication is a recognized limitation of observational comparative effectiveness research and can bias unadjusted associations in either direction, depending on baseline imbalances and treatment-selection mechanisms [17]. In addition, the monotherapy subgroup was relatively small, widening confidence intervals and increasing the likelihood of type II error. Therefore, the most defensible interpretation of the comparative results is that they are hypothesis-generating and should be read in the context of randomized evidence rather than as a competing estimate of treatment effect. Beyond randomized data, real-world outcomes with FTD/TPI plus bevacizumab have been summarized in a recent systematic review and meta-analysis, providing supportive aggregated evidence from observational cohorts while also highlighting the heterogeneity and methodological limitations inherent to non-randomized comparisons [18].

To improve interpretability, we performed a multivariable Cox proportional hazards model for overall survival, adjusting for key baseline prognostic factors (ECOG performance status, metastatic burden, liver metastasis, and age). In this adjusted model, treatment group was not significantly associated with overall survival. In contrast, ECOG performance status and liver metastasis emerged as independent adverse prognostic factors, which is consistent with clinical experience and guideline-based risk stratification in metastatic colorectal cancer [4]. These findings reinforce that, in later-line mCRC, patient fitness and disease distribution frequently dominate outcomes and should guide shared decision-making, monitoring intensity, and early toxicity management when initiating oral cytotoxic therapy.

The apparent divergence between the direction of the univariable treatment hazard ratio and adjusted estimate should be interpreted cautiously. In modestly sized cohorts with substantial baseline imbalances, treatment-effect estimates can be sensitive to model specification and correlated prognostic covariates, even when adjustments are clinically sensible [17]. We considered additional propensity-based adjustment approaches, including propensity score matching, inverse probability weighting, and propensity-adjusted Cox models. However, given the modest cohort size, the relatively small monotherapy subgroup, and the degree of imbalance across several clinically relevant baseline variables, we judged these methods to be at substantial risk of unstable estimates, limited overlap, and overfitting. Rather than implying a true reversal of therapeutic effect, this pattern underscores the limitations of non-randomized comparisons and the importance of transparent framing: the adjusted analysis here serves to contextualize observed survival patterns, not to establish causality.

Tumor response outcomes in our evaluable subset were modest, which is expected in a refractory population where objective responses are uncommon and disease stabilization is often the main achievable goal of treatment. This aligns with the broader late-line mCRC landscape, in which survival gains can occur despite low objective response rates [4, 6, 16]. In our evaluable subset, the combination group showed a numerically higher proportion of partial responses, whereas the monotherapy group showed a higher proportion of stable disease. Thus, the higher ORR observed with combination therapy and the higher DCR observed with monotherapy do not necessarily represent contradictory efficacy signals; rather, they reflect different distributions across response categories in a small retrospective subset. In real-world settings, response assessment is further influenced by heterogeneous imaging schedules and documentation practices, which can lead to variability in response categorization and progression timing. Consequently, response and PFS findings should be interpreted as supportive descriptive signals rather than precise comparative endpoints.

Safety outcomes in our cohort were consistent with the established toxicity profile of FTD/TPI-based therapy. Hematologic toxicity and fatigue were frequent, and severe neutropenia occurred at rates broadly consistent with those reported previously [6, 11, 12, 16]. Treatment discontinuation, where documented, was driven predominantly by disease progression rather than toxicity in both groups (Supplementary Table S2). This is clinically important because maintaining dose intensity in later lines of treatment often depends on timely recognition of cytopenias, structured blood count monitoring, and proactive dose modifications. The absence of unexpected safety signals supports the feasibility of FTD/TPI-based therapy in Turkish routine practice when access is available.

The molecular characterization of the cohort should also be interpreted with caution. Missing MSI/MMR and HER2 results likely reflect historical testing practices, reimbursement constraints, and incomplete retrospective documentation across centers, rather than the true biological absence of these biomarkers.

A key contribution of this study is its region-specific perspective. Access to FTD/TPI has historically been constrained in Turkey; therefore, late-line sequencing can differ from settings in which both regorafenib and FTD/TPI are widely available and routinely interchangeable based on patient factors. In guidelines, both agents are recognized as later-line options, and the choice is typically individualized based on prior exposures, performance status, comorbidities, and toxicity considerations [4, 7]. Real-world evidence from settings with access limitations provides additional context for how evidence-based regimens are implemented in practice and may help clinicians and stakeholders interpret external trial results within local healthcare-system realities. Although the generalizability of these findings is limited by the retrospective design, modest cohort size, and regional practice setting, the study adds clinically relevant real-world evidence from a non-Asian population that remains comparatively underrepresented in the FTD/TPI literature.

Taken together, our results indicate that FTD/TPI-based therapy can be delivered in Turkish routine practice with outcomes broadly consistent with international experience. At the same time, observational comparisons between combination therapy and monotherapy remain strongly influenced by baseline imbalance, non-standardized progression assessment, and residual confounding.

**Fig. 1:**
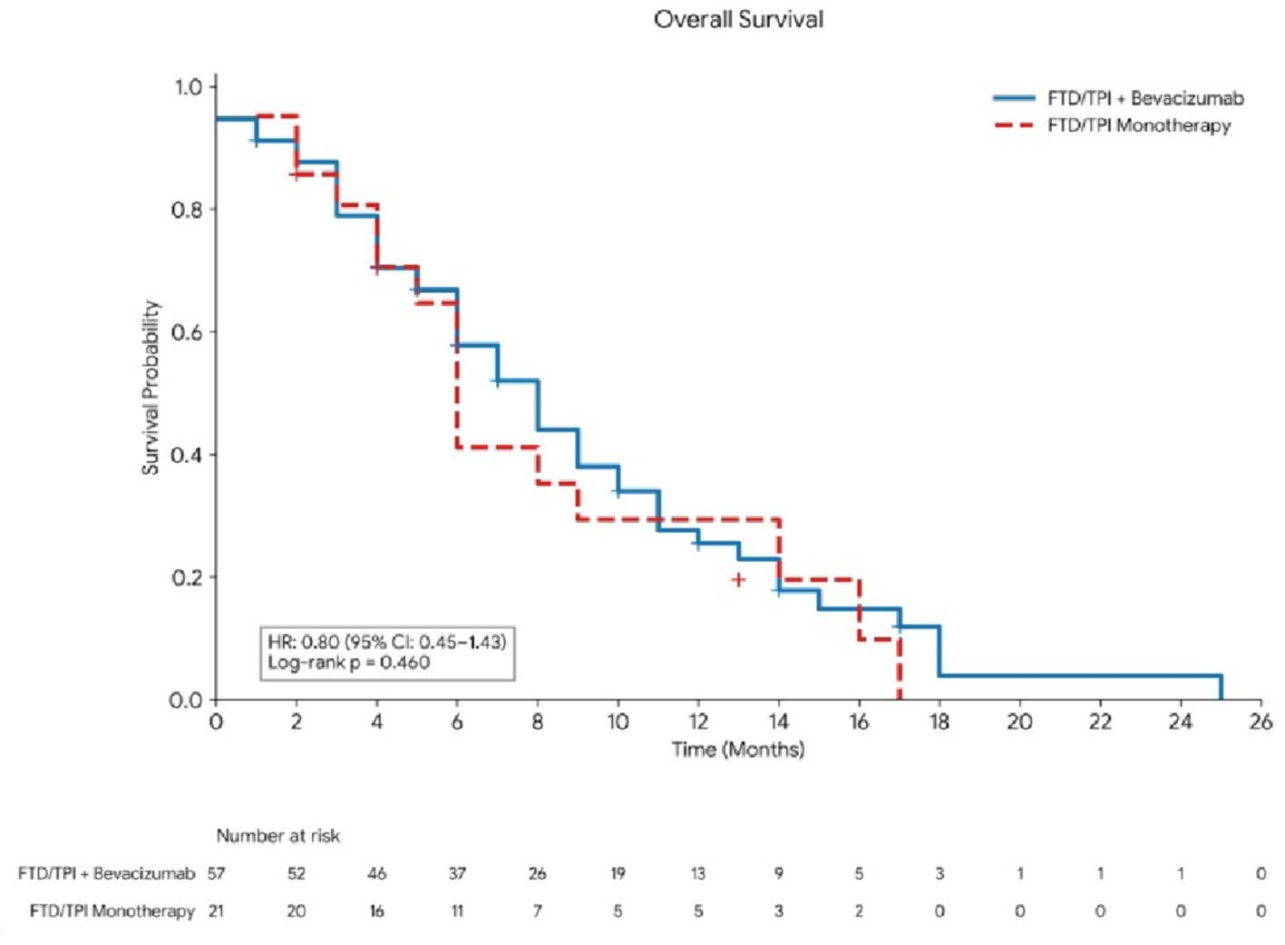
Kaplan–Meier curves for the overall survival (OS) of patients in the FTD/TPI plus bevacizumab group vs. the FTD/TPI monotherapy group. OS, overall survival; FTD/TPI, trifluridine/tipiracil; Bev, bevacizumab; HR, hazard ratio; CI, confidence interval

**Fig. 2:**
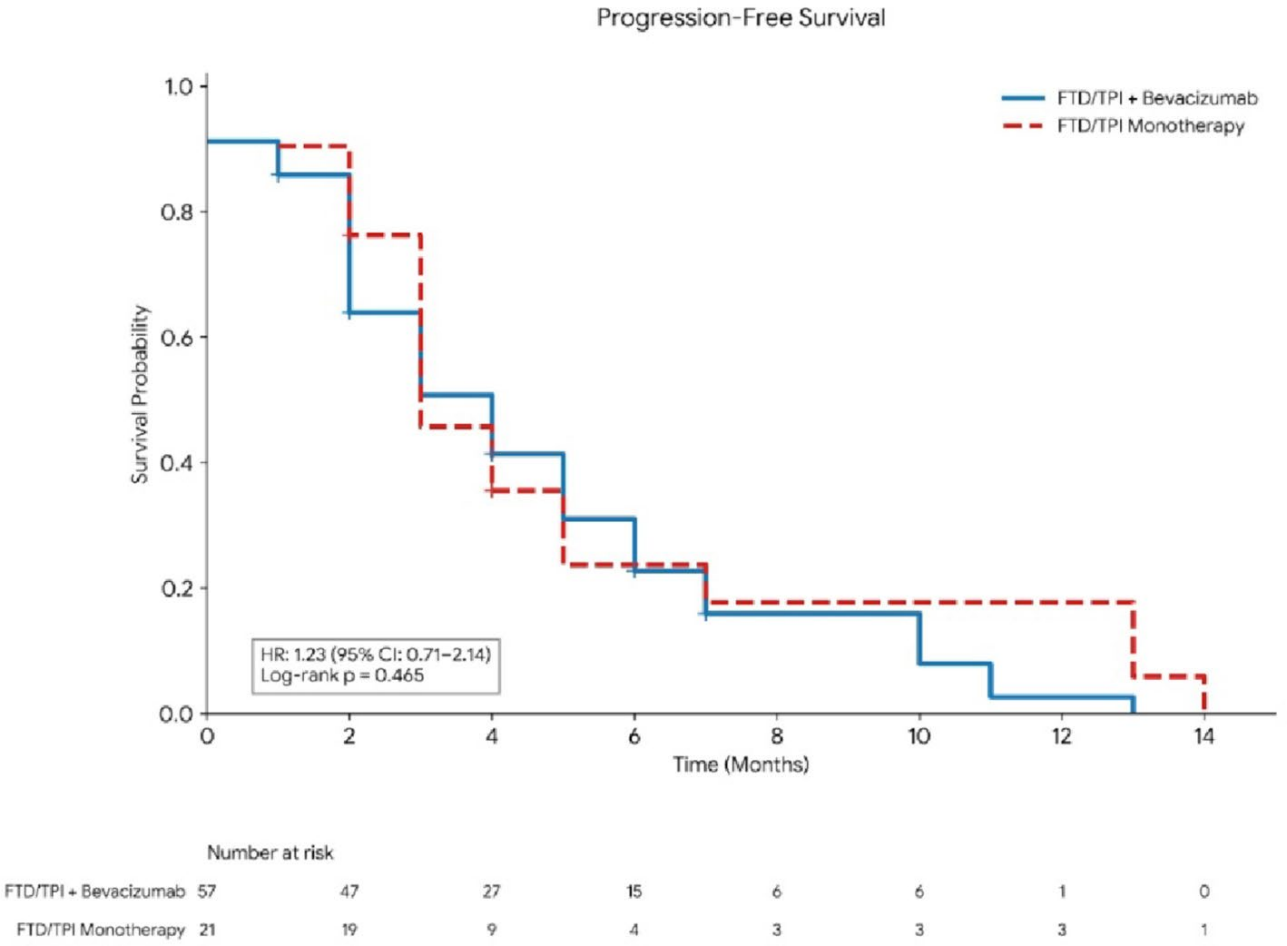
Kaplan–Meier curves for the progression-free survival (PFS) of patients in the FTD/TPI plus bevacizumab group vs. the FTD/TPI monotherapy group. PFS, progression-free survival; FTD/TPI, trifluridine/tipiracil; Bev, bevacizumab; HR, hazard ratio; CI, confidence interval

Therefore, our findings primarily inform feasibility, safety, and practice patterns in a real-world setting, while the comparative efficacy of FTD/TPI plus bevacizumab should continue to be interpreted in light of randomized evidence (Figs. 1, 2).

### Limitations

This study has several limitations inherent to its multicenter retrospective observational design. Treatment allocation was physician-directed rather than randomized, introducing substantial risk of selection bias and confounding by indication. Baseline imbalances between treatment groups, particularly with respect to ECOG performance status, metastatic burden, non-regional lymph node metastasis, and prior treatment exposure, limit the interpretability of comparative estimates. Although a prespecified multivariable Cox model was used to adjust for key prognostic factors, residual confounding from unmeasured or incompletely measured variables cannot be excluded, and the adjusted estimates should not be interpreted causally. The total cohort was modest in size, and the monotherapy subgroup was relatively small, reducing statistical power, widening confidence intervals, and increasing the risk of unstable regression estimates and type II error. Outcome assessment reflected routine practice without centralized radiologic review or standardized imaging intervals, which may have affected PFS and response classification. Adverse events were captured retrospectively and may have been underreported or incompletely graded; moreover, some bevacizumab-specific toxicities, such as hypertension, bleeding, and proteinuria, were not documented with sufficient consistency for robust pooled comparison. Detailed treatment-exposure variables, including exact FTD/TPI cycle counts and the total number of bevacizumab administrations, were not available in a sufficiently standardized format across centers. Although treatment discontinuation reason was available for a substantial subset of patients and was driven predominantly by progression, these data were not complete for the entire cohort. Finally, missing biomarker data for MSI/MMR and HER2 likely reflect historical testing practices, access and reimbursement variability, and incomplete retrospective documentation. Accordingly, these findings should be interpreted as descriptive real-world evidence and confirmed in larger prospective or systematically collected observational datasets.

## Conclusions

In this multicenter Turkish real-world cohort of heavily pretreated patients with metastatic colorectal cancer, FTD/TPI-based therapy was feasible and demonstrated a safety profile broadly consistent with established experience. Although OS and PFS numerically favored FTD/TPI plus bevacizumab, these differences were not statistically significant and should be interpreted as exploratory in light of the retrospective design, baseline imbalances, subgroup-size asymmetry, and residual confounding. The main contribution of this study is to provide region-specific real-world evidence on the implementation and tolerability of FTD/TPI-based therapy in routine practice. These findings should therefore be viewed as complementary to, rather than competitive with, the comparative efficacy estimates established in randomized trials such as SUNLIGHT.

## Supplementary Information

Supplementary Material 1

## Data Availability

The datasets generated and/or analyzed during the current study are not publicly available due to institutional data protection policies but are available from the corresponding author upon reasonable requests.
