# Supplementary material for: Real-world outcomes of trifluridine/tipiracil with or without bevacizumab in refractory metastatic colorectal cancer: a multicenter cohort study from Turkey
Source: Discov Oncol. 2026 May 8;17:963. doi: 10.1007/s12672-026-05158-y (PMC13323716; doi:10.1007/s12672-026-05158-y)
Supplement: Supplementary file 1 — Supplementary Material 1 [file 12672_2026_5158_MOESM1_ESM.docx]

**Supplementary Material**

**Supplementary Table S2. Documented treatment discontinuation reasons by treatment group**

| Discontinuation reason | Combination therapy, n/N (%) | Monotherapy, n/N (%) |
| --- | --- | --- |
| Disease progression | 43/44 (97.7) | 16/17 (94.1) |
| Adverse event / toxicity | 1/44 (2.3) | 1/17 (5.9) |

Footnote: Treatment discontinuation reason was available only for patients with documented coding in the retrospective dataset. Coding definition: 0 = progression; 1 = adverse event/toxicity. Percentages are calculated among patients with available discontinuation data in each treatment group.
